# Adherence, Perception of, and Attitude toward Influenza and Flu Vaccination: A Cross-Sectional Study among a Population of Future Healthcare Workers

**DOI:** 10.3390/ijerph182413086

**Published:** 2021-12-11

**Authors:** Emanuele Chittano Congedo, Maria Emilia Paladino, Michele Augusto Riva, Michael Belingheri

**Affiliations:** 1 School of Medicine and Surgery, University of Milano-Bicocca, 20900 Monza, Italy; e.chittanocongedo@campus.unimib.it (E.C.C.); maria.paladino@unimib.it (M.E.P.); 2 Occupational Health Service, University of Milano-Bicocca, 20126 Milan, Italy

**Keywords:** influenza vaccine, flu vaccination campaign, healthcare students, healthcare workers, occupational medicine

## Abstract

Healthcare students (HCSs) represent a target category for seasonal flu vaccination. This study aimed to examine adherence to flu vaccination campaigns from 2016 to 2019 among HCSs and to investigate knowledge and perception of and attitude toward influenza and flu vaccination. This cross-sectional study was conducted among the HCSs of a northern Italian university. Data on adherence, knowledge, perception, and attitude were investigated through an anonymous online self-administered questionnaire. The questionnaire was filled out by 352 out of 392 third-year HCSs (response rate = 90%). The main reason for refusal was the perception of influenza as non-threatening (24.4%), while self-protection was the main reason for adherence (87.5%). A univariate logistic regression analysis revealed some statistically significant associations with the adherence to the 2018–2019 campaign: being a nursing/midwifery student (OR: 4.14; 95% CI: 1.77–9.71) and agreeing with (OR: 19.28; 95% CI: 2.47–146.85) or being undecided (OR: 10.81; 95% CI: 1.33–88.27) about the obligation of vaccination in health facilities. The associations were also evaluated with a multiple logistic regression model. Despite the low vaccine uptake, good knowledge of the risks for HCSs and patients related to flu has emerged. Improving promotion strategies will be necessary to increase the adhesion of future healthcare workers.

## 1. Introduction

During traineeships in hospitals and other health facilities, healthcare students (HCSs) are at risk of contracting seasonal influenza and transmitting it to patients and healthcare workers (HCWs), playing an important role in nosocomial outbreaks.

Influenza viruses can be transmitted via droplet spread, air, and direct contact. They usually cause an acute respiratory and systemic illness, characterized by fever, asthenia, myalgia, arthralgia, and upper airway inflammation. In most cases, the clinical picture is self-limiting and resolves within about a week, but life-threatening complications may also occur. Worldwide, seasonal influenza every year causes 3 to 5 million cases of severe illness and about 290,000 to 650,000 deaths [1], while in Italy, the cases are 6 to 8 million and the deaths are on average 8,000 [2].

Vaccination represents one of the most effective measures against seasonal influenza and it is highly recommended to HCWs and HCSs by the WHO [3], the CDC [4], and the Ministry of Health of Italy [5] in order to avoid nosocomial outbreaks. Moreover, seasonal influenza is one of the main causes of absenteeism and disruption of health services during the colder months, a period notoriously marked by an increase in demand for healthcare [6].

Nowadays, flu vaccination is even more important, considering that it is almost certain that influenza viruses will circulate in conjunction with SARS-CoV-2 [7]. Furthermore, symptoms caused by both viral infections are usually similar, making differential diagnosis even more difficult. Moreover, the consequences of co-infection are still unclear, considering also that new strains of these two viruses may spread [8].

Despite the ease of access to flu vaccination, which is usually free for HCWs and HCSs, and its scientifically demonstrated effectiveness in reducing the incidence of infection, its acceptance is still a critical issue [9]. In Italy, as stated in the last technical report of ECDC about influenza vaccination coverage rates in the EU/EEA [10], the percentage of HCWs who get vaccinated is far from the goal of 75%, which is set by the Italian National Vaccination Prevention Plan [5].

Unfortunately, data on vaccination coverage among HCSs are not available either at the national or at the regional level. The problem of their poor adherence to flu vaccination campaign has emerged in recent years, since HCSs represent an important target group. In literature, there are only few studies conducted in Italy on this population, mainly among medical and nursing students [11,12,13], while HCSs of other degree courses are not usually considered.

In this context, the purpose of this study was to evaluate the adherence to influenza vaccination campaigns among HCSs of a university in northern Italy, according to their degree course. Another objective was to investigate the knowledge and perception of and attitude toward influenza and flu vaccination among the same population, composed only of third-year students, combining these variables with the number of administered vaccinations.

## 2. Materials and Methods

### 2.1. Design and Subjects

A cross-sectional study was conducted among all students enrolled in the nine degree courses of the school of Medicine and Surgery of a medium-sized university in northern Italy: medicine and surgery, dentistry, nursing, physiotherapy, midwifery, dental hygiene, biomedical laboratory techniques, imaging and radiotherapy techniques, and childhood neuro- and psychomotricity.

### 2.2. Methods

We conducted a survey consisting in the self-administration of an anonymous online questionnaire by third-year HCSs between January 2019 and March 2019. Participation was voluntary, free of compensation, and completely anonymous since no personal data were collected during the survey.

The questionnaire was created with Google Forms, provided freely by Google Inc. Questions were based on a survey derived from a systematic review of the literature on the topic [14], formulated by a group of experts (including a vaccinologist, a research nurse, and a public health specialist) [6].

The questionnaire was composed of three different sections. The first section collected information about the degree course attended, age, gender, self-reported adherence to previous flu vaccination campaigns (2016–2017, 2017–2018, and 2018–2019 seasons), reasons for accepting or refusing it, and intention to be vaccinated the following year.

The second section investigated students’ agreement or disagreement with some statements about vaccinations, in order to assess their knowledge and perception of and attitude toward influenza and flu vaccine. In detail, the questions investigated students’ awareness about the national ministerial recommendations for influenza vaccine, its safety and efficacy, and nosocomial risks related to influenza virus. Finally, this section investigated participants’ perception of the interference of pharmaceutical companies in health policies, the role of health professionals in encouraging the immunization of colleagues, and new strategies to improve vaccination uptake.

The third section included questions about the specific flu vaccination campaigns that the Occupational Health Service organizes every year. This part investigated the respondents’ awareness about the possibility of receiving free flu shots at the hospital, opinions about the hospital’s ad campaign for flu vaccination, and suggestions to increase adherence to the flu vaccination campaign.

### 2.3. Data Analysis

Data were analyzed using the statistical software SAS 9.4 (SAS Institute, Cary, NC, USA), and the level of statistical significance was set at *p* < 0.05. All variables considered in this study were categorical, expressed as absolute and relative frequencies. The chi-square test or Fisher’s exact test were used to compare two frequencies in case of low sample numbers.

A univariate logistic regression model was used to evaluate the association between dependent variables (adherence to the vaccination campaign) and independent variables (gender, degree, and agreement or disagreement with a series of statements in the questionnaire). Values of odds ratio (OR) and 95% confidence interval (95% CI) were reported. The associations were also evaluated with a multiple logistic regression model. To further correct the analyses for multiple comparisons, we also applied the Bonferroni correction.

## 3. Results

### 3.1. Adherence to Flu Vaccination Campaigns

The questionnaire was filled out by 352 out of 392 third-year HCSs (response rate = 90%). Participants were from different courses: medicine and surgery (32.1%), nursing (25.3%), physiotherapy (11.4%), dental hygiene (7.1%), childhood neuro and psychomotricity (7.1%), dentistry (5.9%), imaging and radiotherapy techniques (4.3%), midwifery (3.4%), and biomedical laboratory techniques (3.4%). Since some degree courses were small in number, they were aggregated into three major categories, in order to increase the statistical power: medicine and surgery and dentistry, nursing and midwifery, and other degree courses.

Students were largely female (66.8%), the average age was 22.4 years, and the standard deviation was ±2.4.

As regards the adherence to flu vaccination campaigns, 45 students (12.8%) declared to have been vaccinated in the 2016–2017 season, 29 students (8.2%) in the 2017–2018 season, and 48 students (13.6%) in the 2018–2019 season. Table 1 shows the distribution of participants by degree course and the flu vaccination coverage in the 2018–2019 season.

### 3.2. Reasons for Accepting or Refusing Flu Vaccination

The reasons for accepting or refusing flu vaccination are reported in Table 2 and Table 3, respectively. Among the 48 students who were favorable to the vaccination, the main reasons were to protect themselves (87.5%), protect patients (56.3%), and protect friends and family (52.1%). On the contrary, the main reasons related to refusing the flu vaccine were the opinion that flu is not a dangerous disease (24.4%), the lack of awareness of the possibility of getting vaccinated in the hospital, and the lack of time to get vaccination (20.0%).

Finally, 36.6% of respondents claimed that they were willing to be vaccinated in the following year, while the remaining participants were divided almost equally between the unwilling (31.0%) and the undecided (32.4%).

### 3.3. Data on Knowledge and Perception of and Attitude toward Influenza and Flu Vaccination

Table 4 reports the results of students’ agreement with some statements related to knowledge and perception of and attitudes toward flu vaccination and influenza. Most of the participants agreed that they can get flu vaccination easily (90.1%), health professionals have a higher risk of contracting flu (87.5%), and flu vaccination is safe (85.8%). Furthermore, most of the responders were not concerned about local or systemic reactions to flu vaccination (74.5%) or about the side effects of vaccination (73.9%). About half of the students did not know the recommendations about the vaccination during pregnancy (46.9%).

We performed a univariate logistic regression analysis to assess the association between the variables collected and the adherence to vaccination in the 2018–2019 season, including gender, degree course, and agreement with the statements in Table 4. The results of this analysis are presented in Table 5.

In univariate analysis, the adherence to the 2018–2019 flu vaccination campaign was associated with female gender (OR: 2.77; 95% CI: 1.25–6.13) and being a nursing and midwifery student (OR: 4.91; 95% CI: 2.32–10.38). Agreeing with some statements related to flu vaccination was associated with the adherence to the flu vaccination campaign, such us the opinion that flu is a potentially dangerous disease, that health professionals should get flu vaccination every year, and that health professionals have a role in promoting the vaccination among colleagues and patients (Table 5).

The results of multiple logistic regression between the variables collected and the adherence to vaccination in the 2018–2019 season are shown in Table 6.

The analysis confirmed that being a nursing and midwifery student was associated with the adherence to the flu vaccination campaign (OR: 5.33; 95% CI: 1.89–15.02), while concerns about the side effects of vaccination were associated with a decreased adherence to the flu vaccination campaign (OR: 0.06; 95% CI: 0.01–0.46). However, this last association was weakened when we applied the Bonferroni correction for multiple comparisons.

Finally, a further univariate logistic regression analysis was performed to compare the adherence to the 2018–2019 vaccination campaign with the intention to get vaccinated the following year. Vaccinated students had a greater chance of getting vaccinated even in the following year (OR: 39.75; 95% CI: 12.03–131.41) (data not shown).

### 3.4. Data on the Promotion of Flu Vaccination Campaign and Improvement Strategies

The answers to the questionnaires showed that 53.7% of the respondents were aware of the possibility of receiving free flu vaccination at the Occupational Health Service. Students were informed by teachers (24.1%), other students (18.5%), leaflets posted in universities and in the hospital (18.2%), and hospital staff (9.1%). A few students were informed by social networks (5.4%) or emails sent directly from the university (1.1%).

However, 33.0% of the participants considered the advertising campaign sufficient and 34.1% poor. About 6.8% of the respondents considered it insufficient. On the contrary, 21.6% of the students declared that the ad campaign was good and 4.5% found it excellent.

Finally, students were asked how to increase vaccine uptake the following year. The answers were organizing an informative lecture on the importance of flu vaccination (57.4%); vaccinating students directly in the university (45.7%); vaccinating students directly in hospital during internships (42.6%); and promoting the vaccination campaign more effectively through social networks, emails, and leaflets (35.8%).

## 4. Discussion

Our study aimed at evaluating the adherence to influenza vaccination campaigns among HCSs and investigating their knowledge and perception of and attitude toward influenza and flu vaccination. The choice to administer the questionnaire only to third-year students of all degree courses was made to compare HCSs of almost the same age. However, this could represent a selection bias, since medical and dentistry students were still in the middle of their six-year academic program and had just started their traineeships. Therefore, they were probably less aware of the importance of vaccination and the possible consequences of their non-adherence. On the contrary, the others are three-year degree courses, in which traineeships begin the first year. Consequently, their students have more clinical experience and awareness of the importance of flu vaccination. Even in the literature, it is reported that students who have attended several traineeships are more likely to get vaccinated than those who do not have a wide clinical experience yet [15,16]. Another reason as a possible explanation of inhomogeneous results in the prevalence of vaccinations is the role of specific topics that could not be similarly covered in different courses and different years, such as microbiology, infectious diseases, health hygiene, and occupational medicine. However, courses such as medicine and surgery or dentistry should have better coverage of these topics since the courses last six years, but our results showed less adherence to the flu vaccination campaign among medical students than students of other courses.

Among the reasons to get vaccinated (Table 2), a predominantly individualistic approach has emerged, while only approximately half of the sample got vaccinated to protect patients, friends, and family.

As for the reasons for refusing the vaccination, it is worrying that almost a quarter of the students believed that flu is not potentially dangerous, since they are supposed to be aware of the potential complications of this disease. Moreover, the fact that they were not aware of the possibility of being vaccinated is evidently due to an ineffective promotion. Organizational problems, such as forgetting to get vaccinated and the lack of time, could be related to the elevated work and study load.

The perception of vaccination by most students was positive (Table 4), as it was considered safe by 85.6% of respondents and effective by 61.3%. In addition, about 74.0% stated that they were not worried about side effects and local or systemic reactions and 90.1% thought they could get the vaccine easily. These high percentages are comforting and demonstrate a positive perception of vaccination, probably due to an adequate knowledge of flu vaccination, its safety, and its limited side effects. Only one student declared to be against vaccination.

It is also positive that 53.4% thought they could play a role in promoting vaccination among friends and patients: indeed, word of mouth among students was an effective means of promotion, as shown in the results section.

As far as knowledge about influenza and flu vaccine is concerned, the answers are encouraging. In fact, the majority of the participants were aware that influenza is a potentially dangerous disease (66.8%), that it can lead to epidemics (82.1%), and that the risk of contracting influenza is greater for healthcare workers (87.5%), who should be vaccinated every year (65.6%).

However, this positive response is in stark contrast to the percentage of adhesion: only 13.6% of the respondents. In fact, although 66.8% declared to be aware of the potential danger of influenza, it was not considered a dangerous disease by the majority of those who had not been vaccinated. This demonstrates the need to emphasize to the people of concern in future vaccination campaigns the dangerousness of this disease and at the same time the importance of vaccination for themselves and others.

Just half of the sample was aware of the recommendation of flu vaccination for pregnant women, and only 28.1% knew the ministerial recommendations. It would be advisable to discuss these aspects in more detail during lectures in order to increase these percentages.

Half of the sample agreed with the introduction of mandatory vaccination in healthcare facilities and 37.2% with the obligation to make unvaccinated people wear surgical masks during the flu season, initiatives that could significantly reduce the spread of the influenza virus.

As already highlighted in Table 6, only two factors were independently associated with adherence to the 2018–2019 influenza vaccination campaign, so the strength of association of most of these variables was lost. In fact, although many more associations were found through the univariate logistic regression analysis, these were not confirmed at the multiple logistic regression. This is probably due to the insufficient size of our sample, as only 48 out of the 352 students reported as having been vaccinated during the 2018–2019 season.

The first independently associated factor was the degree course: the probability of being vaccinated for nursing and midwifery students was almost five times higher than for medical students, probably due to their greater clinical experience, as assumed before.

The concerns about the side effects of vaccination were associated with a decreased adherence to the flu vaccination campaign. Although few students were concerned about the side effects of vaccination, it is important to consider this topic, which can be properly addressed by providing students with information about flu vaccine side effects. However, the association between side effects and a decreased adherence to the vaccination campaign was weakened when we applied the Bonferroni correction for multiple comparisons.

It is noteworthy that HCSs who joined the 2018–2019 vaccination campaign were more likely to be vaccinated the following year than those who did not join it. This finding is in accordance with the study conducted by De Juanes et al. concerning adherence to three consecutive vaccination campaigns in a population of HCWs [17]. It showed that those who had been vaccinated in previous campaigns were four to nine times more likely to adhere to future ones.

Finally, the fact that only 53.7% of the respondents were aware of the possibility of receiving free flu vaccination at the hospital was definitely correlated to the low adherence. After the administration of the questionnaire, the importance of vaccination was reiterated to the students and more details were given about when and how to get vaccinated. In this way, since the response rate among the HCSs was equal to 90%, it was possible to promote the campaign to almost all third-year students.

As regards how respondents were informed about the possibility of being vaccinated, the answers to the questionnaire showed that promotion via social networks and emails sent by the university was not effective. The most effective publicity was by leaflets posted in universities and in the hospital, by teachers, and by word of mouth among students. Since the publicity of the campaign was evaluated negatively by more than 40% of the sample, it is recommended to strengthen all these promoting means in order to reach as many students as possible.

The adherence to vaccination could also increase by implementing the other strategies proposed at the end of the questionnaire. In particular, 57.4% of the respondents were in favor of organizing informative lectures on the importance of flu vaccination, held by teachers and doctors at the Occupational Health Service, possibly with compulsory attendance to ensure the participation of as many students as possible. Moreover, almost half of the students were in favor of getting vaccinated directly at the university or during traineeships. Efforts should, therefore, be made to implement both strategies in the future, with the collaboration of hospital staff, with the aim of making access to vaccination easier.

This study has some potential limitations. The choice to administer the questionnaire only to third-year students of all degree courses could represent a selection bias, since medical and dentistry students were still in the middle of their six-year academic program and had just started their traineeships. Consequently, these students could have less clinical experience and awareness of the importance of flu vaccination. A second limit of the study is the small sample size and the small number of students who got the vaccination.

Another limitation of this study consists in the use of a self-administered questionnaire, in which participants may minimize their opinion against vaccination. However, the survey was anonymous and the participation was voluntary, so students may have answered honestly. Nevertheless, recent studies have shown that this method of data collection has a high sensitivity and moderate specificity, especially if the respondents are adults in good health [18,19].

## 5. Conclusions

This study helped to better understand adherence and rejection factors regarding flu vaccination among HCSs. Low vaccination uptake was mainly due to the perception of influenza as a non-threatening disease and the weak promotion of vaccination campaigns, elements that need to be actively worked on in order to increase the coverage rate.

In conclusion, it is important to underline the fact that education of healthcare workers about seasonal flu vaccination should start when they are still students, in order to consolidate this habit. In fact, once they become healthcare workers, they will be required to promote the vaccine to patients. Therefore, encouraging HCSs to get vaccinated represents the first step toward an increase in vaccination uptake in the entire community. These considerations could be also applied to the recent COVID-19 pandemic, since HCSs should be considered as healthcare workers and the reasons of adherence to COVID-19 vaccination campaigns should be known [20,21,22].

## Figures and Tables

**Table 1:** Distribution of participants and students who were vaccinated.

Degree Course	Students Who Filled Out the Questionnaire *N* (%)	Students Who Were Vaccinated in the 2018–2019 Season *N* (%)
Medicine and surgery/Dentistry	134 (38.1)	11 (8.2)
Nursing and midwifery	101 (28.7)	31 (30.7)
Other degree courses	117 (33.2)	6 (5.1)
Total	352 (100)	48 (13.6)

**Table 2:** Reasons for accepting flu vaccination (*n* = 48).

	Answers *N* (%)
**I want to protect myself from the flu.**	42 (87.5)
**I want to protect patients from the flu.**	27 (56.3)
**I want to protect friends and family from the flu.**	25 (52.1)
**Flu vaccine is free.**	16 (33.3)
**I do not want to lose days of lectures or internship.**	8 (16.7)
**I want to follow the recommendations of the Ministry of Health.**	7 (14.6)
**My friends also got vaccinated.**	0 (0.0)

**Table 3:** Reasons for refusing flu vaccination (*n* = 304).

	Answers *N* (%)
**I think flu is not a dangerous disease.**	74 (24.4)
**I was not aware of the possibility of getting vaccinated in the hospital and/or I was never offered.**	67 (22.1)
**I did not have time to get vaccinated.**	61 (20.0)
**I was going to get vaccinated, but I forgot to do it.**	59 (19.5)
**I do not think I am adequately informed about the utility of the flu vaccination.**	48 (15.8)
**I have never had the flu.**	36 (11.9)
**I think that flu vaccination does not work or does not provide effective protection.**	21 (6.9)
**I am afraid of needles.**	16 (5.3)
**I am afraid of side effects.**	9 (3.0)
**I could not get vaccinated because at that time I was sick.**	5 (1.7)
**I am against vaccinations.**	1 (0.3)

**Table 4:** Agreement or disagreement with statements about flu vaccination and influenza.

	I Agree *N* (%)	I Disagree *N* (%)	I Do Not Know *N* (%)
Flu vaccination is safe.	302 (85.8)	6 (1.7)	44 (12.5)
Flu vaccination is effective.	216 (61.4)	26 (7.4)	110 (31.2)
I can get flu vaccination easily.	317 (90.1)	0 (0.0)	33 (9.9)
I am concerned about the side effects of vaccination.	42 (11.9)	260 (73.9)	50 (14.2)
I am concerned about local or systemic reactions to flu vaccination.	42 (11.9)	262 (74.5)	48 (13.6)
Health professionals have a higher risk of contracting flu.	308 (87.5)	10 (2.8)	34 (9.7)
Health professionals should get flu vaccination every year.	231 (65.6)	32 (9.1)	89 (25.3)
Flu is a potentially dangerous disease.	235 (66.8)	60 (17.0)	57 (16.2)
Flu can cause epidemics in hospitals.	289 (82.1)	9 (2.6)	54 (15.3)
I think I can play a role in promoting the vaccination among colleagues and patients.	188 (53.4)	39 (11.1)	125 (35.5)
I believe that flu vaccination should be mandatory in healthcare settings.	174 (49.4)	66 (18.8)	112 (31.8)
I believe that wearing surgical masks in hospital settings during the flu season should be mandatory for unvaccinated people.	131 (37.2)	98 (27.8)	125 (35.0)
I know the ministerial recommendations regarding the prevention of influenza.	99 (28.1)	121 (34.4)	132 (37.5)
I believe that pharmaceutical companies influence decisions on vaccination strategies.	99 (28.1)	117 (33.3)	136 (38.6)
Flu vaccination is recommended for pregnant women.	177 (50.3)	10 (2.8)	165 (46.9)

**Table 5:** Univariate logistic regression analysis of the variables associated with the adherence to vaccination during the 2018–2019 campaign.

Variable	OR	95% CI
**Gender**		
Male	Ref.	
Female	2.77	1.25–6.13
**Degree course**		
Medicine and surgery/Dentistry	Ref.	
Nursing and midwifery	4.91	2.32–10.38
Other degree courses	0.60	0.21–1.67
**Flu vaccination is effective.**		
Disagree	Ref.	
Agree	1.49	0.42–5.24
Do not know	0.77	0.19–3.01
**Flu is a potentially dangerous disease.**		
Disagree	Ref.	
Agree	3.60	1.07–12.10
Do not know	2.61	0.64–10.65
**Health professionals have a higher risk of contracting flu.**		
Disagree	Ref.	
Agree	1.46	0.18–11.85
Do not know	1.20	0.12–12.13
**Health professionals should get flu vaccination each year.**		
Disagree	Ref.	
Agree	7.54	1.00–56.72
Do not know	0.71	0.06–8.14
**Flu vaccination is recommended for pregnant women.**		
Disagree	Ref.	
Agree	1.41	0.17–11.63
Do not know	1.47	0.18–12.12
**I am concerned about the side effects of vaccination.**		
Disagree	Ref.	
Agree	0.40	0.12–1.35
Do not know	0.33	0.10–1.11
**I am concerned about local or systemic reactions to flu vaccination.**		
Disagree	Ref.	
Agree	0.79	0.29–2.14
Do not know	0.68	0.25–1.83
**I think I can play a role in promoting the vaccination among my colleagues and patients.**		
Disagree	Ref.	
Agree	10.01	1.33–75.18
Do not know	2.26	0.31–21.43
**I believe that pharmaceutical companies influence decisions on vaccination strategies.**		
Disagree	Ref.	
Agree	0.94	0.43–2.07
Do not know	1.01	0.50–2.08
**I know the ministerial recommendations regarding the prevention of flu.**		
Disagree	Ref.	
Agree	3.63	1.73–7.61
Do not know	0.58	0.23–1.49
**Flu can cause epidemics in hospitals.**		
Disagree	Ref.	
Agree	0.62	0.12–3.06
Do not know	0.21	0.03–1.46
**I believe that flu vaccination should be mandatory in health facilities.**		
Disagree	Ref.	
Agree	16.48	2.21–123.00
Do not know	7.80	0.99–61.43
**I believe that wearing surgical masks in hospital facilities during the flu season should be mandatory for unvaccinated people.**		
Disagree	Ref.	
Agree	1.97	0.89–4.34
Do not know	1.11	0.47–2.61

**Table 6:** Factors associated with adherence to the 2018–2019 flu vaccination campaign resulting from multiple logistic regression analysis.

Variable	OR	95% CI
**Degree course**		
Medicine and surgery/Dentistry	Ref.	
Nursing and midwifery	5.33	1.89–15.02
Other degree courses	0.46	0.12–1.71
**I am concerned about the side effects of vaccination.**		
Disagree	Ref.	
Agree	0.06	0.01–0.46
Do not know	0.29	0.04–2.51

## Data Availability

The data presented in this study are available on request from the corresponding authors. The data are not publicly available due to privacy reason.
